# Evaluating the knowledge, attitude, and practices among youth regarding HIV transmission, its prevention, and their stigmatizing attitude toward people living with HIV: an exploratory cross-sectional study

**DOI:** 10.1097/MS9.0000000000005245

**Published:** 2026-06-16

**Authors:** Hafiza Ayesha Ghersheen, Ayesha Azeem, Sumia Fatima, Arthi Roy, Sadeem Arshad, Ritaba Arooj, Subhania Ehsan, Zoya Batool Satti, Warda Fatima Shafee, Muddassir Khalid

**Affiliations:** a Department of Medicine, Bolan Medical College, Quetta, Pakistan; b Department of Medicine, Rawalpindi Medical University, Rawalpindi, Pakistan; c Department of Medicine, Pabna Medical College, Pabna, Bangladesh; d Department of Medicine, Nishtar Medical University, Multan, Pakistan

**Keywords:** acquired immunodeficiency syndrome, adolescent, AIDS-related opportunistic infections, attitude, diagnosis, fomites, HIV, patients, prevalence, social discrimination

## Abstract

**Objective:**

This study assessed the knowledge of the youth of Pakistan regarding HIV transmission, their misconceptions, and their stigmatizing attitudes toward people living with HIV/AIDS in an exploratory cross-sectional sample.

**Methods:**

A cross-sectional study was conducted at Rawalpindi Medical University; the duration of the study was 6 months, from May to October (2025). Our sample size was 308. Data were collected using a questionnaire. The sampling technique was non-random convenience sampling, and data analysis was performed using SPSS version 28 with chi-square tests.

**Results:**

This study included 308 participants, with 62.3% females (*n* = 192) and 37% males (*n* = 114), divided into three age groups: 15–19 years (8.8%, *n* = 27), 20–24 years (57.5%, *n* = 177), and 25–29 years (31.2%, *n* = 96). Regarding HIV transmission knowledge, 91.9% (*n* = 283) correctly identified sexual intercourse as a mode, whereas misconceptions existed – 5.2% (*n* = 16) believed HIV could spread via a handshake, and 15.6% (*n* = 48) thought it could be transmitted through mosquito bites, indicating good overall awareness with some gaps. Stigmatization was notable: 22.1% (*n* = 68) felt uncomfortable around people with HIV/AIDS, 13.1% (*n* = 40) would quit their job rather than work with an HIV-positive person, 26% (*n* = 80) would not hug someone with HIV/AIDS, and 33.4% (*n* = 104) associated HIV/AIDS with declining moral values. A significant association was found between misconceptions and negative attitudes (*P* < 0.001), suggesting a lack of knowledge.

**Conclusion:**

Pakistani youth demonstrated strong knowledge of common HIV transmission routes but held critical misconceptions that contribute to stigmatizing attitudes. Enhancing education and open dialog on HIV prevention and treatment is essential to improving awareness and reducing stigma, thereby fostering public support for people living with HIV/AIDS.

## Introduction

HIV, a lentivirus from the Retroviridae family, targets CD4+ T lymphocytes, impairing the immune system and leading to acquired immunodeficiency syndrome (AIDS)^[^1^]^. First identified in 1983, HIV has claimed 40.4 million lives up to 2022. In 2019, 36.9 million people had HIV/AIDS globally (0.5% of the world population), with rising prevalence in South Africa, Portugal, Brazil, Mexico, Peru, Spain, Germany, and the United States^[^2^]^. According to 2023 data, about 39.9 million people live with HIV, 1.3 million were newly infected, and 630,000 died from HIV-related causes worldwide^[^3^]^.

HIGHLIGHTS

- Nationwide assessment of HIV knowledge, attitudes, and practices among Pakistani youth.
- High awareness of sexual transmission, with persistent misconceptions identified.
- Misconceptions strongly associated with stigmatizing attitudes toward people living with HIV.
- One-third of participants linked HIV with moral decline and social exclusion.
- Findings support targeted youth education to reduce HIV-related stigma.

Pakistan reported its first HIV case in 1987 due to an unsafe blood transfusion, with outbreaks among intravenous drug users in 2004 and mass infections in Kot Imrana village in 2018, which had a 13% prevalence^[^4,5^]^. The 2021 HIV/AIDS Data Hub registered approximately 210 000 HIV cases in Pakistan, mostly among adults, with fewer cases than in neighboring countries^[^6^]^.

HIV transmits through bodily fluids such as blood, semen, breast milk, and via contaminated medical equipment^[^7^]^. Despite this, 63% globally lack adequate knowledge about transmission, and 76% hold misconceptions^[^8^]^. Earlier seen mainly in at-risk groups, HIV/AIDS stigma persists globally, linked to ignorance and cultural factors, causing social isolation and mental health issues in people living with HIV (PLHIV)^[^9,10^]^. Common myths include transmission via mosquito bites (70.4%) and casual contact^[^11^]^.

Preventive programs like the ABC strategy (abstinence, faithfulness, and condom use) reduced HIV prevalence from 30% in the early 1990s to 6.4% by 2005, but prevalence slightly rose later due to behavioral and socioeconomic factors^[^12,13^]^. Despite progress, knowledge gaps and stigma remain significant, especially among Pakistani youth, underscoring the need for targeted education and awareness campaigns to promote early diagnosis, prevention, and reduce stigma to curb the HIV epidemic effectively.

“This cross-sectional study has been reported in line with the STROCSS guidelines [Agha R, Mathew G, Rashid R, Kerwan A, Al-Jabir A, Sohrabi C *et al* Revised Strengthening the Reporting of Cohort, Cross-Sectional, and Case-Control Studies in Surgery (STROCSS) Guideline: An Update for the Age of Artificial Intelligence. Premier Journal of Science, 2025]” (25).

## Materials and methods

This descriptive, exploratory, cross-sectional study was conducted over 6 months, from May to October, among youth aged 15–29 years at Rawalpindi Medical University. The sample size of 308 was calculated using the Cochrane formula, allowing for a 95% confidence interval with a 0.05 margin of error, using a *P*-value of 0.5 from the parent article^[^1^]^. Non-random convenience sampling was used to recruit participants, including males and females who had not been previously diagnosed with HIV/AIDS. Exclusion criteria comprised individuals who did not complete the consent form, those outside the age range, those with a prior HIV/AIDS diagnosis, and those with cognitive, psychological, or communicative impairments preventing questionnaire completion.

Data collection was performed using a bilingual (Urdu and English) questionnaire adapted from prior research ^[^2,3,6^]^ to ensure clarity and inclusivity. It contained closed-ended demographic questions, including age and gender, along with items evaluating knowledge, misconceptions, and stigma related to HIV/AIDS. Youth understanding of HIV transmission was assessed by six statements (e.g., HIV transmission through sexual intercourse or breastfeeding), with responses scored as 1 (yes), 0 (no), and “I do not know” responses treated as neutral. The mean score was 3, indicating greater knowledge when met or exceeded. Misconceptions were assessed via five statements (e.g., transmission by handshaking), scored similarly, with a mean of 2.5 reflecting more prevalent myths when higher.

Stigma toward PLHIV/AIDS was measured using 14 items (e.g., unwillingness to hug someone with AIDS), with yes/no/maybe answers scored as 1/0/0. The mean score was 7; scores above this indicated stigmatizing attitudes, while those below reflected positive attitudes. Prevention behaviors were assessed by seven Likert-scale items (always/sometimes/never scored as 3/2/1), with a mean score of 3 indicating full awareness and practice of preventive measures.

Following ethical approval and informed consent, questionnaires were distributed in educational institutions and community areas. Responses were anonymized and kept confidential, with completed forms securely archived for analysis. Data were analyzed using SPSS version 28, employing descriptive statistics and chi-square tests for associations.

## Results

This exploratory study assessed knowledge, misconceptions, prevention awareness, and stigmatizing attitudes regarding HIV among Pakistani youth. The sample consisted of 308 participants: 62.3% females (*n* = 192) and 37% males (*n* = 114; Table 1). Participants were divided into three age groups: 15–19 years (8.8%, *n* = 27), 20–24 years (57.5%, *n* = 177), and 25–29 years (31.2%, *n* = 96; Table 1).

**Table 1:** Demographic variables.

Variables	Percentages
Gender
Female	62.3%
Male	37%
Age
15–19 years	8.8%
20–24 years	57.5%
25–29 years	31.2%

Regarding knowledge of HIV transmission, 91.9% (*n* = 283) correctly identified sexual contact as a transmission mode, 95.1% (*n* = 293) recognized transmission via infected needles/syringes, and 90.9% (*n* = 280) were aware of blood transfusion risks. Awareness of vertical transmission was lower at 65.6% (*n* = 202), with only 56.5% (*n* = 174) recognizing that vertical transmission is preventable (Table 2).

**Table 2:** Knowledge about HIV transmission.

Statement	Percentages
HIV transmission by sexual relation
Yes	91.9%
No	9.1%
HIV transmission by infected syringes and needles:
Yes	95.1%
No	4.9%
HIV transmission by blood transfusion:
Yes	90.9%
No	9.1%
HIV can be transmitted from mother to child during pregnancy and labor:
Yes	65.6%
No	34.4%
Transmission of HIV through breastfeeding
Yes	48.4%
No	51.6%

Misconceptions were notable: 5.2% (*n* = 16) believed transmission occurs via handshake, 15.6% (*n* = 48) via mosquito bites, 6.5% (*n* = 20) via hugging, while a large 50.6% (*n* = 156) thought saliva could transmit HIV. Additionally, 34.4% (*n* = 106) believed sharing utensils spread HIV, and 48.4% (*n* = 149) thought breastfeeding could transmit the virus. Chi-square analysis showed significant gender differences in misconceptions: females exhibited more misconceptions overall, except that males had more misconceptions regarding transmission via mosquito bites and saliva (*P* < 0.001, α = 0.005; Table 3).

**Table 3:** Chi square relation between variables.

		*P*-value shows the significant relation between gender and misconceptions.
Transmission of HIV by handshake		*P* < 0.001
Yes	5.2%	
No	94.8%	
Transmission of HIV through mosquito bite		*P* < 0.001
Yes	15.6%	
No	84.4%	
Transmission of HIV by hugging an infected person		*P* < 0.001
Yes	6.5%	
No	93.5%	
Transmission of HIV by sharing utensils		*P* < 0.001
Yes	34.4%	
No	65.6%	
Transmission of HIV through saliva of an infected person		*P* < 0.001
Yes	51.6%	
No	49.4%	

Stigmatizing attitudes were prevalent: 22.1% (*n* = 68) felt uneasy around people with AIDS, 13.1% (*n* = 40) would quit their jobs rather than work with someone infected, and 26% (*n* = 80) refused to hug an HIV-positive person. Notably, 42.2% (*n* = 130) stated their support depends on how someone contracted HIV (e.g., extramarital sex or multiple partners). Furthermore, 9.7% (*n* = 30) believed PLHIV should blame themselves, and 33.4% (*n* = 104) associated AIDS with moral decline. Negative perceptions included 24% (*n* = 74) who opposed PLHIV having children, 40.6% (*n* = 125) who felt HIV transmission should be punishable by law, and 56.2% (*n* = 173) who believed PLHIV should not be sexually active. Over half (51.9%, *n* = 160) would prevent their children from playing with HIV-positive children, and 36.7% (*n* = 113) refused to buy vegetables from PLHIV. Additionally, 21.4% (*n* = 66) would not allow an HIV-positive teacher to continue teaching. Moreover, 51.6% (*n* = 159) attributed rising HIV rates to greater sexual freedom. Statistical analysis confirmed that misconceptions significantly correlate with negative attitudes toward PLHIV (*P* < 0.001, α = 0.005), indicating that poor knowledge drives stigma (Table 4).

**Table 4:** Social myths and stigmas surrounding AIDS.

Statements	Percentages
Does being around with person having AIDS bother you
* Yes*	22.1%
* No*	49%
* May be*	28.2%
Would quit my job if I had to work with person living with AIDS
* Yes*	13%
* No*	69.8%
* May be*	16.6%
I would not hug someone with AIDS
* Yes*	26%
* No*	52.3%
* May be*	26%
My support for the person living with AIDS depends upon how they got AIDS
* Yes*	42.2%
* No*	42.9%
* May be*	14.3%
People with AIDS only have themselves to blame
* Yes*	9.7%
* No*	73.7%
* May be*	15.9%
Spread of AIDS is linked to the decline of moral values
* Yes*	33.4%
* No*	32.55
* May be*	33.4%
Women who know they are infected with AIDS have the right to have children
* Yes*	46.8%
* No*	24%
* May be*	28.6%
Transmitting AIDS virus should be punishable by law
* Yes*	40.6%
* No*	32.1%
* May be*	26.6%
People who know they have AIDS, if they spread it intentionally then they are criminals
* Yes*	53.9%
* No*	26%
* May be*	19.5%
People with AIDS have the right to be sexually active
* Yes*	18.8%
* No*	56.2%
* May be*	24.4%
If a teacher has HIV/AIDS should he/she be allowed to teach in the school
* Yes*	62%
* No*	21.4%
* May be*	15.9%
Arrival of the AIDS is linked to the fact that people have more sexual freedom
* Yes*	51.6%
* No*	22.7%
* May be*	25%

Regarding prevention, 28.2% (*n* = 87) actively sought reliable HIV/AIDS information (“always”), 51% (*n* = 157) did so “sometimes,” and 20.1% (*n* = 62) “never.” When encouraging family/friends to attend awareness sessions, 43.2% (*n* = 133) “always,” 28.6% (*n* = 88) “sometimes,” and 27.6% (*n* = 85) “never” encouraged participation. Conversations about HIV/AIDS with friends were “always” held by 15.9% (*n* = 49), “sometimes” by 51.9% (*n* = 160), and “never” by 31.5% (*n* = 97). Family discussions were less frequent: 9.7% (*n* = 30) “always,” 39.9% (*n* = 123) “sometimes,” and 49.7% (*n* = 153) “never” (Table 5).

**Table 5:** Strategies to reduce social stigma.

Statements	Percentages
How often do you actively seek out reliable information regarding HIV transmission, its prevention from reputable resources
Always	28.2%
Sometimes	51%
Never	20.1%
How often do you encourage your family/friends to participate in programs providing awareness regarding HIV transmission and its prevention
Always	43.2%
Sometimes	28.6%
Never	27.6%
How often do you talk to your friend regarding HIV and its prevention
Always	15.9%
Sometimes	51.9%
Never	31.5%
How of do you talk to your family members regarding HIV and its prevention
Always	9.7%
Sometimes	39.9%
Never	49.7%
How often do you engage in practices that reduces the risk of HIV and its prevention
Always	43.8%
Sometimes	23.7%
Never	31.8%
How often do you perform self-examination to check any abnormal changes in person living with HIV/AIDS
Always	22.1%
Sometimes	38.6%
Never	38.6%
How often do you use sterile needle and syringes when needed
Always	61.2%
Sometimes	7.1%
Never	37%

In terms of risk reduction behaviors, 43.8% (*n* = 135) “always” engaged in prevention activities (e.g., limiting partners, condom use, sterile syringes), 23.7% (*n* = 73) “sometimes,” and 31.8% (*n* = 98) “never.” Self-examinations related to reproductive health were reported “always” by 22.1% (*n* = 68), “sometimes” by 38.6% (*n* = 119), and “never” by 38.6% (*n* = 119). Finally, 61.2% (*n* = 172) consistently used sterile needles/syringes, 7.1% (*n* = 22) “sometimes,” and 31.7% (*n* = 114) “never.”

## Discussion

The purpose of this exploratory study was to assess youth awareness and misconceptions regarding HIV transmission, their knowledge related to its prevention, and their stigmatizing attitudes toward PLHIV/AIDS in Pakistan. The study population consisted of 308 individuals aged 15–29 years. The majority of the participants were females (62.3%). Most participants were in the 20–24 year age group (57.5%).

Awareness was high among our participants regarding the common modes of HIV transmission, including sexual contact (91.9%), infected syringes and needles (95.1%), and blood transfusion (90.9%). However, a significant minority was not aware of less recognized modes of transmission, including breastfeeding and vertical transmission, and many were also unaware that vertical transmission is preventable. A similar finding was reported in a study conducted in Jordan, where 46% of the population were aware that vertical transmission is preventable, and 41% were aware of transmission through breastfeeding^[^1^]^.

Misconceptions were also present in society regarding HIV transmission: 51.6% believed that HIV could be transmitted by saliva, 15.6% believed in transmission through mosquito bites, 5.2% through handshakes, 6.5% through hugging, and 34.4% through sharing utensils. For instance, in Iran, nearly half of the population mistakenly believed HIV could be transmitted through sneezing, and a significant portion thought mosquito bites could spread the virus^[^2,14,15^]^.

Misconceptions were also related to gender; most were observed among females, except for misconceptions regarding HIV transmission through mosquito bites and saliva, where males were more misinformed. This point highlights the need for personalized education for both genders.

According to our findings, a sizable portion of the population has stigmatizing attitudes toward those who are HIV/AIDS positive. There was a significant correlation between stigmatizing attitudes and misconceptions, as many people hold negative attitudes toward individuals living with HIV/AIDS due to a lack of knowledge about the virus and its modes of transmission. Chi-square analysis and supporting studies confirmed a significant relationship between stigmatizing attitudes and misconceptions. One study carried out in the United Arab Emirates found that this unfavorable attitude resulted from misconceptions^[^3^]^. According to the same study carried out in the United Arab Emirates, HIV is seen as improper (haram) in orthodox society and is solely linked to sexual encounters that are forbidden^[^3^]^.

A study carried out in Iran found that young people dislike interacting with individuals who have HIV/AIDS, kissing them, and sharing their belongings with them^[^2^]^. Another study claims that, in 1998, the Supreme Court of Canada ruled that an individual with AIDS should face legal action if they failed to disclose the disease to others^[^5^]^.

Conversely, some studies demonstrate changing societal attitudes toward PLHIV/AIDS. One study conducted in Ghana indicated a decline in stigmatizing attitudes due to increased understanding of HIV transmission^[^4^]^. However, another study carried out in Iran found that the majority of pupils stated they would not leave their school because of their HIV-positive classmates^[^6^]^. In a similar vein, 85% of female students in a study conducted in Japan said they would stick with their peers^[^7^]^. According to the findings of our study and previous research, societal attitudes toward PLHIV/AIDS vary, and these attitudes largely depend on individuals’ knowledge regarding the modes of HIV transmission.

Lastly, we examined preventive behaviors in society. Encouragingly, many participants were involved in preventive measures: 43.8% reported reducing risk through condom use and sterile injections, 61.2% used sterile needles, while only 28.2% actively sought reliable information regarding HIV, and few encouraged awareness among family and friends. This suggests that educational initiatives have proven effective. A study conducted in the Netherlands among Turkish and Moroccan immigrants reported that community members were trained to educate others about the prevention and transmission of AIDS, which reduced discrimination and misinterpretation^[^8^]^. According to a study conducted among African Americans, the media also plays a crucial role in educating people about HIV transmission and reducing stigmatizing attitudes; the study reported that stigma decreased within 3 months with the help of media^[^9,16,17^]^.

The main cause of these misconceptions and stigmatizing attitudes toward PLHIV/AIDS, according to a study conducted in Indonesia, was a lack of education and knowledge. The study also emphasized the importance of secondary education regarding antiretroviral therapy, or the treatment of HIV, noting that educated people are aware of the availability of antiretroviral therapy and how to treat HIV^[^10^]^. These points highlight the need for comprehensive education regarding HIV transmission and prevention among youth to empower them to adopt preventive measures and help reduce stigmatizing attitudes.

### Limitations

This study used a non-random convenience sampling strategy; therefore, the sample may not accurately represent the broader population of Pakistani youth. As an exploratory cross-sectional study, the findings should not be interpreted as nationally representative, and the results should be generalized with caution. Future studies using probability-based sampling and larger samples across diverse regions of Pakistan are needed to obtain nationally representative estimates. Equal representation of males and females should be ensured.

## Conclusion

In conclusion, Pakistani youth showed significant knowledge of the common modes of HIV transmission. However, regarding the less common modes of transmission, there was a critical gap, and misconceptions regarding HIV transmission were also significant, contributing to a negative attitude. All of this was linked to a lack of education regarding the modes of transmission. Strengthening education and encouraging open discussions on HIV prevention and treatment is mandatory to increase their awareness. It is also necessary to change this negative attitude and make the public more supportive toward PLHIV/AIDS.

## Data Availability

Data available upon request from the authors.
